# A hybrid deep learning and rule-based model for smart weather forecasting and crop recommendation using satellite imagery

**DOI:** 10.1038/s41598-025-21506-4

**Published:** 2025-10-15

**Authors:** Salma A. Mohamed, Olfat O. Abdel Maksoud, Abdelrahman Fathy, Ahmed S. Mohamed, Khaled Hosny, Hatem M. Keshk, Sayed A. Mohamed

**Affiliations:** 1 Faculty of Computers Science and Technology, Modern academy, Cairo, Egypt; 2 National Authority for Remote Sensing and Space Science, Cairo, Egypt

**Keywords:** Smart agriculture, Artificial intelligence, Climate change, Satellite images processing, Climate sciences, Environmental sciences

## Abstract

The effective management of meteorological forecasting data is crucial for enhancing agricultural sustainability and precision, especially considering climate change. This study presents an innovative framework that integrates multispectral image analysis, advanced weather forecasting, and rule-based models to improve agricultural practices in Egypt’s Al-Sharkia region, specifically targeting rice and wheat cultivation. The framework employs artificial intelligence and sophisticated data processing techniques to analyze information from satellites, remote sensing devices, and meteorological stations, delivering accurate weather predictions and climate forecasts. The Convolutional Neural Network (CNN) model classified agricultural land into appropriate categories, exhibiting exceptional performance with a reduction in training loss from 0.2362 to 6.87e-4. The Recurrent Neural Network and Long Short-Term Memory (RNN-LSTM) model demonstrated significant predictive accuracy, achieving a root mean square (RMS) error of 0.19 in forecasting critical meteorological variables. In contrast to prior research that utilizes solely remote sensing or meteorological data, this study introduces an innovative hybrid framework that amalgamates CNN-based image analysis, LSTM-based weather prediction, and rule-based crop advisories. This comprehensive method provides precise, localized forecasts and customized agricultural advice, facilitating informed decisions regarding crop selection, planting schedules, and resource allocation. This thorough methodology, validated by Sentinel-2 and NOAA data, aims to reduce crop losses, decrease operational costs, and encourage sustainable agricultural practices in response to climate change problems.

## Introduction

 The implementation of precision and sustainability in agriculture is contingent upon the intelligent administration of meteorological data. It is a critical component of a variety of duties, including irrigation scheduling, crop growth, development, and yield. Technological advancements have enabled the collection of this meteorological data from a variety of sources at a low cost and with high temporal resolution. Recent studies highlight the growing impact of climate change on agriculture, particularly through its adverse effects on crop yields and soil health^1,2^. These changes not only threaten annual agricultural productivity but also place severe financial strain on farmers. Faced with decreased yields due to climate-induced damage, many farmers are turning to increased fertilizer use as a way to counterbalance their losses. However, this solution brings its own challenges^3^. The recognition of these challenges faced by farmers and the deteriorating health of agricultural land has underscored the need for systematic solutions to mitigate each issue^4^. At the core of these problems is climate change, a fundamental driver of rising temperatures, erratic weather patterns, and shifting growing conditions^5,6^. Research has shown that climate change significantly contributes to increased temperatures, which in turn affects soil quality, water availability, and crop viability. This escalating threat not only impacts individual farmers but also poses risks to the broader agricultural sector, emphasizing the importance of developing adaptive strategies to sustain productivity and protect livelihoods^7–9^. Prolonged periods of extreme high temperatures can have fatal effects on crops, with global temperatures projected to rise between 2 °C and 4.5 °C by the end of this century (IPCC, 2007)^10^. This shift in climate is already impacting crop production on a large scale, with future climate patterns expected to exacerbate these challenges, further threatening agricultural productivity and food security^11^.

The impact of climate change on agriculture in Egypt has been extensively studied using crop models and climate scenarios^12,13^. Most research in this area relies on two primary scenarios—a temperature rise of 1.5 °C and a rise of 3.6 °C—projected to 2050^14–16^. This research focuses on four key climate factors that directly influence crop productivity in Egypt: temperature, humidity, atmospheric pressure, and rainfall. By analyzing these factors with climate forecasting models, scientists can predict future trends and the potential impact on Egyptian agriculture^17^. This predictive approach is essential for developing adaptive strategies and planning measures to mitigate the impacts of climate change, thereby enhancing the resilience and sustainability of Egypt’s agricultural sector^18,19^.

In traditional methods research, several basic factors were studied to estimate the impact of climate change on agriculture^20,21^. Normalized Different Vegetation Index (NDVI), It is used to determine the green plant density in the studied area and reflects the plant growth and general health of plants^22^. Normalized Different Water Index (NDWI), used to measure water availability in plants and soil, which helps estimate the effects of water shortages on plants^23^. Salinity Index level (SI) reflects the level of salinity in the soil, which is an important factor affecting plant growth and productivity. Green Normalized Difference Vegetation (GNDVI), used to determine vegetation density based on the light reflectance’s of trees and green crops. Enhanced Vegetation Index (EVI), Measures plant health and biological activity, providing a comprehensive understanding of plant growth and environmental impacts. Moisture level (MOISTURE) reflects the moisture content in the soil and air and helps in estimating the water needs of plants and providing it effectively. These factors will provide a comprehensive framework for understanding how climate change will affect the agricultural environment and its productivity^24^.

 The subsequent sections of this article are structured in the following manner. Section 2 provides a concise summary of the study area and remote sensing data set. Section 3 introduces the Proposed Methodology for RNN architecture, CNN architecture and Rule based architecture. In addition, the experiments performed are elucidated. Section 4 provides the result of this proposed model with other techniques and includes detailed analysis of the different elements through ablation tests. Lastly, the conclusions encompass an analysis of the observed model’s effectiveness.

## Related work

Recent advancements in agricultural technologies utilizing machine learning and deep learning models have markedly enhanced crop resilience to extreme climatic circumstances, including heat stress and water scarcity^25–27^. Ongoing investment in the development of drought- and heat-resistant cultivars by genetic enhancement and computer modeling is essential for augmenting farmer production and improving agricultural sustainability. The quality of agricultural soil directly affects productivity, presenting substantial issues in the context of changing climatic circumstances. In Egypt, crops such as rice and wheat, essential for national food security, are especially susceptible. This study primarily examines rice and wheat, evaluating their production effects via satellite-based hyperspectral imaging. identified six fundamental soil factors that affect productivity, highlighting their interrelation and contribution to yield improvement. Adverse weather conditions significantly intensify difficulties to crop productivity, particularly abrupt temperature increases. Climatic factors like temperature, precipitation, solar radiation, and humidity directly affect crop growth; hence, these were chosen for comprehensive modeling Data collected over a 20-year period from Egyptian meteorological stations were utilized. Preliminary forecasting utilizing a fundamental Recurrent Neural Network (RNN) demonstrated significant mistakes, especially in temperature predictions, at times surpassing a 4 °C error margin and exhibiting erroneous temporal sequencing of data^28^.To enhance prediction accuracy, implemented a Long Short-Term Memory (LSTM) network, a sophisticated variety of recurrent neural networks, resulting in markedly improved performance. The LSTM accurately forecasted temperature and humidity, preserving chronological sequence and reflecting daily variations. Validation on broad datasets from many situations corroborated its exceptional accuracy, closely aligning with actual observational data the suggested methodology incorporates Convolutional Neural Networks (CNN), Recurrent Neural Networks (RNN), and Long Short-Term Memory (LSTM) models. Convolutional Neural Networks (CNNs) examine hyperspectral imaging data, extracting essential elements for assessing vegetation indices such as NDVI and EVI, which indicate crop health and productivity levels These qualities are incorporated with 10 meteorological and environmental criteria essential for evaluating agricultural productivity amid climate unpredictability. Following the classification of crops influenced by variable weather patterns and soil health, an LSTM model forecasts forthcoming meteorological circumstances, assessing their effects on crop yield. Forecasted weather parameters, along with comprehensive soil health indicators, enable recommendations for ideal crops (rice and wheat), ensuring maximum yield despite climatic fluctuations.

The contribution of this study listed as follows:

- Mitigate agricultural productivity declines and address land degradation resulting from climate-related issues through strategic planting selections.
- Determine ideal planting periods for rice and wheat by the dynamic analysis of temporal weather patterns and soil health with advanced deep learning techniques.
- Alleviate climate-related production declines by identifying ideal environmental conditions for planting.
- Develop a more comprehensive approach for assessing site suitability and predicting optimal planting times to guarantee sustainable agricultural productivity and adaptability to climate variations.

**Table 1:** Provides a comparison between previous approaches and the current study, highlighting methodological improvements and contributions.

Comparison	Paper 1: Weather Forecasting Using Machine Learning Techniques^29^	Paper 2: Assessing Climate Change Impacts on Crop Yields and Exploring Adaptation Strategies in Northeast China^30^	Paper 3: Enhancing crop recommendation systems with explainable AI^31^	Our Proposed Model
***Name***	Weather Forecasting using Machine Learning Techniques	Weather Impact Analysis on Agriculture Using Remote Sensing and Machine Learning	Climate-Aware Crop Recommendation Using Multispectral and IoT Sensors	Weather Forecasting Model Using LSTM
***Objective***	Forecast temperature and rainfall using time series analysis.	Evaluate climate impact on crop productivity using ML and remote sensing.	Enhancing crop recommendation systems with explainable AI	Develop a hybrid forecasting model for weather and agriculture optimization.
***Algorithm***	Time series using RNN	CNN for spatial data, RNN for temporal data	Decision Trees + SHAP (Explainable ML), Ensemble Model	LSTM for time series, CNN for spatial data, rule-based models for crop selection.
***Data Used***	Meteorological data (12 years, 2006–2018).	Remote sensing imagery and meteorological data (5 years).	Sentinel-2 imagery, soil data, weather data (2020–2023)	Hyperspectral imagery and meteorological data (4 years, 2020–2024).
***Factors Considered***	Temperature and rainfall.	Temperature, rainfall, NDVI, EVI, soil salinity.	Temperature, humidity, soil salinity, NDVI, land use, crop calendar	Temperature, humidity, rainfall, NDVI, NDWI, salinity, moisture level, pressure.
***Geographical Scope***	Single-region study (generalized location).	Multi-regional data across three agricultural zones.	Nile Delta and Middle Egypt (local and regional level)	Al-Sharkia Governorate, Northern Egypt (specific agricultural hotspot).
***RMS Error/Accuracy***	1.41	0.95	Accuracy: 92%, Precision: 89%	0.19
***Computational Complexity***	Moderate	High (due to remote sensing processing).	Medium (Explainable models are lighter than deep nets)	High (complex hybrid architecture).
***Strengths***	Simple time series analysis.	Integrates spatial and temporal data for better accuracy.	Combines accuracy with transparency; useful in real-world deployment	Combines spatial, temporal, and rule-based approaches for optimal crop planning.
***Limitations***	Limited accuracy in long-term forecasts; lacks spatial data.	Moderate error in temperature forecasts; lacks advanced temporal models like LSTM.	Needs large, well-labeled datasets; not as adaptive to highly dynamic changes	Requires high computational resources; limited to areas with sufficient datasets.
***Practical Applications***	Weather forecasting for general agricultural practices.	Assess climate risks for strategic agricultural decision-making.	Crop selection advisory, farmer decision support systems	Specific crop recommendations, climate mitigation strategies, irrigation planning.
***Weather Factors Predicted***	Temperature, Rainfall	Temperature, Humidity, NDVI, Soil Moisture, and other vegetation indices.	Temperature, rainfall, humidity (inputs, not predictions)	Temperature, Humidity, Rainfall, NDVI, NDWI, Soil Moisture, Salinity, Pressure.
***Data Source(s)***	National weather stations and historical weather data.	Remote sensing imagery, weather stations, and ground-based sensors.	Sentinel-2, MODIS, ground-based crop reports	Weather stations, satellite imagery (hyperspectral), ground data sensors.
***Prediction Horizon***	Short-term (up to 5 days).	Short to medium-term (up to 1 year).	Recommendations are event-triggered (no forecast horizon)	Short to medium-term (up to 1 year).
***Model Interpretability***	Moderate (RNN lacks transparency in decision-making).	Moderate to high (CNN and RNN models provide insights into spatial and temporal patterns).	Very High (SHAP and feature importance make the system transparent)	High (LSTM allows for sequence forecasting, and rule-based model adds transparency).

To further illustrate the comparative performance of the models, an aggregated bar chart summarizing key classification metrics is provided below.

The findings are distinguished from recent works such as^32–35^, which focus on isolated data sources or lack integrated forecasting and decision-making layers. By combining CNN-based spatial classification, LSTM-based time-series forecasting, and rule-based crop reasoning, this model provides end-to-end support for precision agriculture. This hybrid approach, supported by strong evaluation metrics (93.5% accuracy, RMSE 0.19), offers a more comprehensive and deployable framework than existing models.

## Materials and methods

This section provides a comprehensive overview of the data, methodologies, and study area utilized in this research. employed satellite imagery, sophisticated deep learning architectures (CNN and RNN-LSTM), and a rule-based classifier to create a comprehensive agricultural decision-making framework. The applied approaches concentrate on the exact assessment of agricultural land suitability and the precise forecasting of weather conditions to inform strategic agricultural actions.

### Proposed methodology

**Fig. 1:**
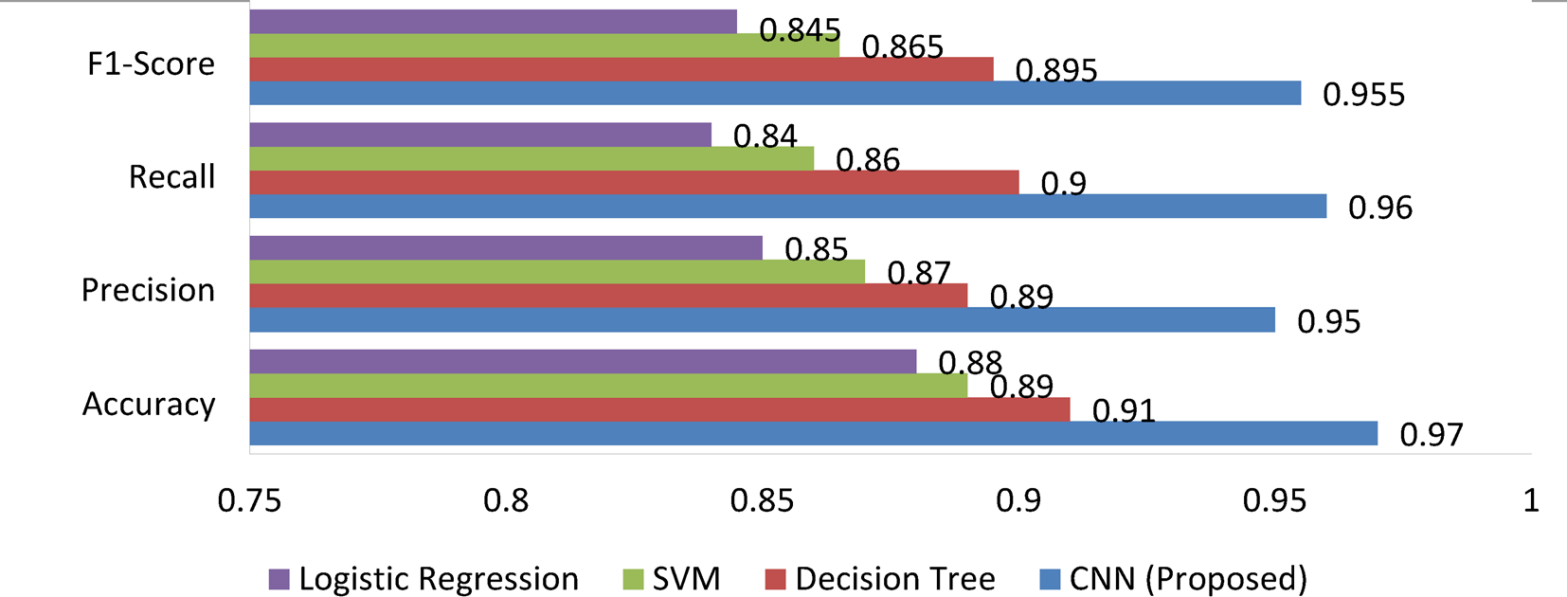
This figure presents a visual performance comparison of the proposed CNN model and baseline classifiers (Decision Tree, SVM, and Logistic Regression) based on four evaluation metrics: Accuracy, Precision, Recall, and F1-Score. The CNN model demonstrates superior results across all metrics, supporting its effectiveness in land suitability classification.

To improve crop management efficiency and attain superior, more productive yields, the suggested methodology presents a novel fusion of multispectral satellite imaging, deep learning, and agro-environmental analysis. Utilizing Sentinel-2 satellite data facilitates accurate monitoring of vegetation health, soil moisture, and nutrient concentrations, along with the prompt identification of diseases and pests. this methodology distinctively integrates dynamic spectral indices with a CNN-based classification model to assess agricultural land suitability at high resolution, in contrast to traditional methods that address spatial and temporal aspects independently. The case study, executed in Egypt’s Al-Sharkia Governorate from 2022 to 2023, employs a statistically rigorous analysis of soil and vegetation attributes. This facilitates the determination of ideal planting and harvesting times based on specific meteorological and spectral patterns. the method creates a representative dataset that accurately reflects the region’s agricultural variability by integrating several spectral features into composite raster layers. This integrated, data-driven methodology signifies a substantial improvement over conventional single-variable studies, facilitating adaptive, site-specific decision-making for sustainable agriculture Fig. 1. 

#### Spectral indices network - convolutional neural networks (CNN)

NDVI (Normalized Difference Vegetation Index), NDWI (Normalized Difference Water Index), Moisture, EVI (Enhanced Vegetation Index), NDSI (Normalized Difference Snow Index), and GNDVI (Green Normalized Difference Vegetation Index) were all evaluated.

Combined use of this data to evaluate the performance of the proposal use Methodology in predicting the factors affecting soil trace agriculture, considering that the temporal pattern of canopy parameters can change between different sites due to changes in local weather conditions across the Al-Sharkia region. open experiments were conducted in Al-Sharkia as Fig. 5, Egypt during the 2022/2023 season. This work aimed to evaluate the performance of agricultural soil and learn from it to create a complete classification of the agricultural land to ensure the soil’s adaptation to climate changes.

These calculations were carried out through following Equations:

1 $$\:\text{N}\text{D}\text{V}\text{I}=\frac{(NIR+Red)}{\:(NIR-Red)\mathbf{}}$$

2 $$\:\text{N}\text{D}\text{W}\text{I}=\frac{(Green-NIR)}{(Green+NIR)}$$

3 $$\:\text{M}\text{o}\text{i}\text{s}\text{t}\text{u}\text{r}\text{e}=\frac{(narrow-swir)}{(narrow+swir)}$$

4 $$\:\text{E}\text{V}\text{I}\hspace{0.17em}=\hspace{0.17em}\text{G}\times\:\frac{(NIR-Red)}{(NIR+C1\times\:Red-C2\times\:Blue+L)}$$

5 $$\:\text{N}\text{D}\text{S}\text{I}=\frac{(Green-SWIR)}{(Green+SWIR)}$$

6 $$\:\text{G}\text{N}\text{D}\text{V}\text{I}=\frac{(NIR-Green)\text{}}{(NIR+Green)\text{}}$$

Each spectrum band represents a certain range of wavelengths as appear in Table 1.

**Fig. 2:**
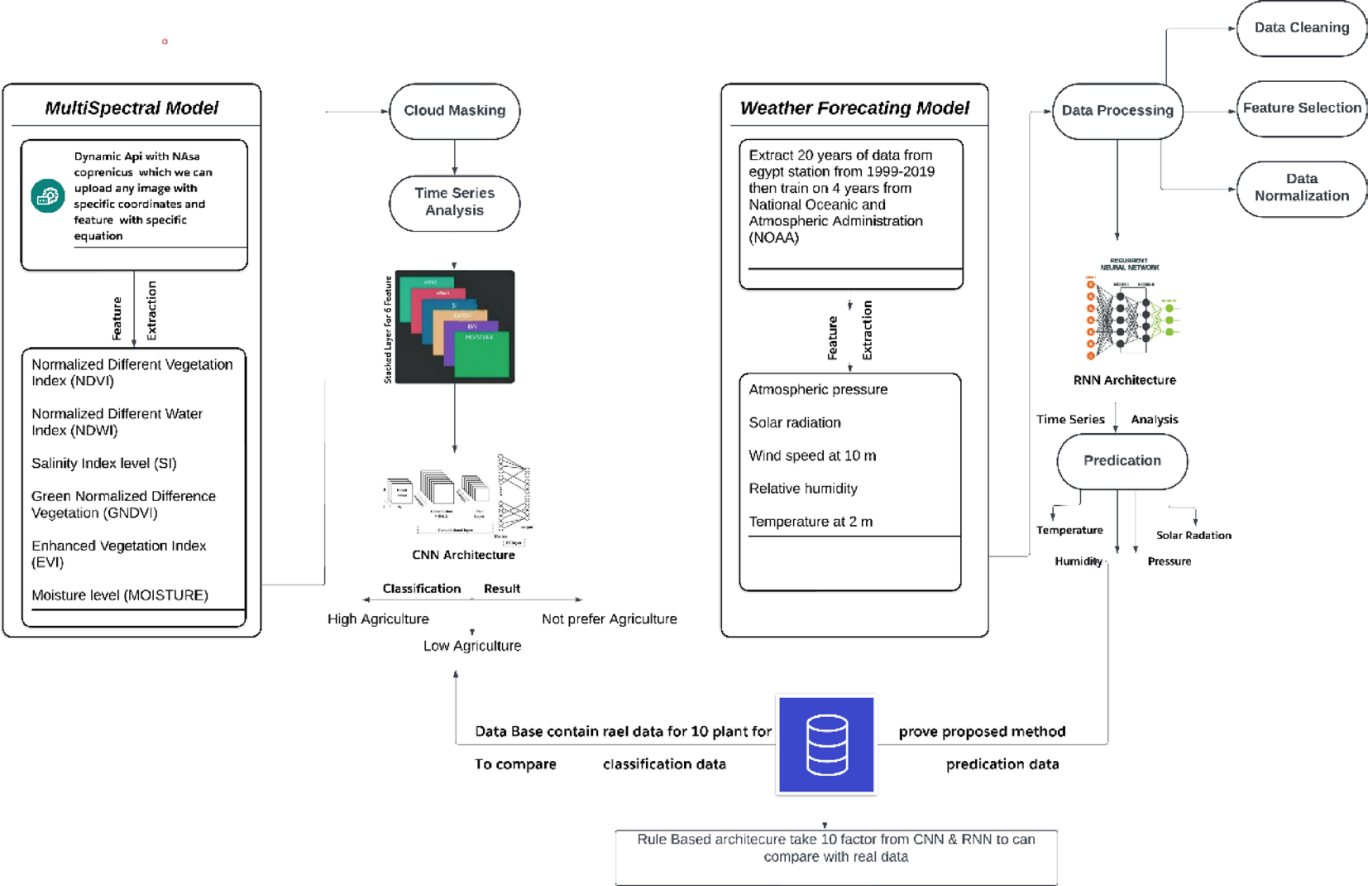
The architecture provides an overview of the methodology for assessing forecasts by means of temperature forecasts and Multispectral image. The flowchart also illustrates the methodology for forecasting by using a complete set of forecasted variables.

Hyperspectral imaging provides accurate spectral information for each pixel in an image by recording several data bands. The identification and characterization of materials through their distinct spectral fingerprints facilitates numerous applications, including medical imaging, agriculture, environmental monitoring, and mineral extraction. This comprehensive construct utilizes a dynamic API from the NASA Copernicus system to extract features dynamically at any specified coordinates, demonstrating the notion of assumption through the application of CNN architecture in unsupervised deep learning prediction as shown in Fig. 2, resulting in raster data images for each feature. Subsequently, each characteristic is transformed into raster data to facilitate the creation of a stacked layer, illustrating the interrelationships among these features and identifying commonalities throughout each area for agricultural land analysis, as it encompasses all pertinent data for the study. The stacked layers facilitate the examination of bands for classification based on particular ranges for each cluster. Which categorizes a given region into: - High agricultural preference - Low agricultural preference - No preference for agriculture.

#### Weather forecasting network - recurrent neural network (RNN)

Forecasts of atmospheric pressure, solar radiation, wind speed at 10 m, relative humidity and temperature at 2 m were acquired from National Oceanic and Atmospheric Administration (NOAA) outputs, with lead times from 1 to 5 days^36^. The HydroMeteoClimate Regional Service’s COSMO model is one of the many small-scale modeling systems used by the National Oceanic and Atmospheric Administration (NOAA). NOAA can give precise and accurate weather and climate predictions because of these cutting-edge modeling techniques^36^. Weather forecasting models are sophisticated computer systems that calculate and predict future weather conditions. These models mimic the behavior of the atmosphere by considering several meteorologically significant factors, such as:

- This is a crucial component in determining wind direction, cloud formation, and precipitation.
- Changes in temperature influence wind direction, precipitation, cloud formation, and the types of clouds that form.
- Humidity affects the likelihood of fog, cloud formation, and precipitation.
- Wind controls cloud and precipitation distribution, wind direction, and wind speed.

**Fig. 3:**
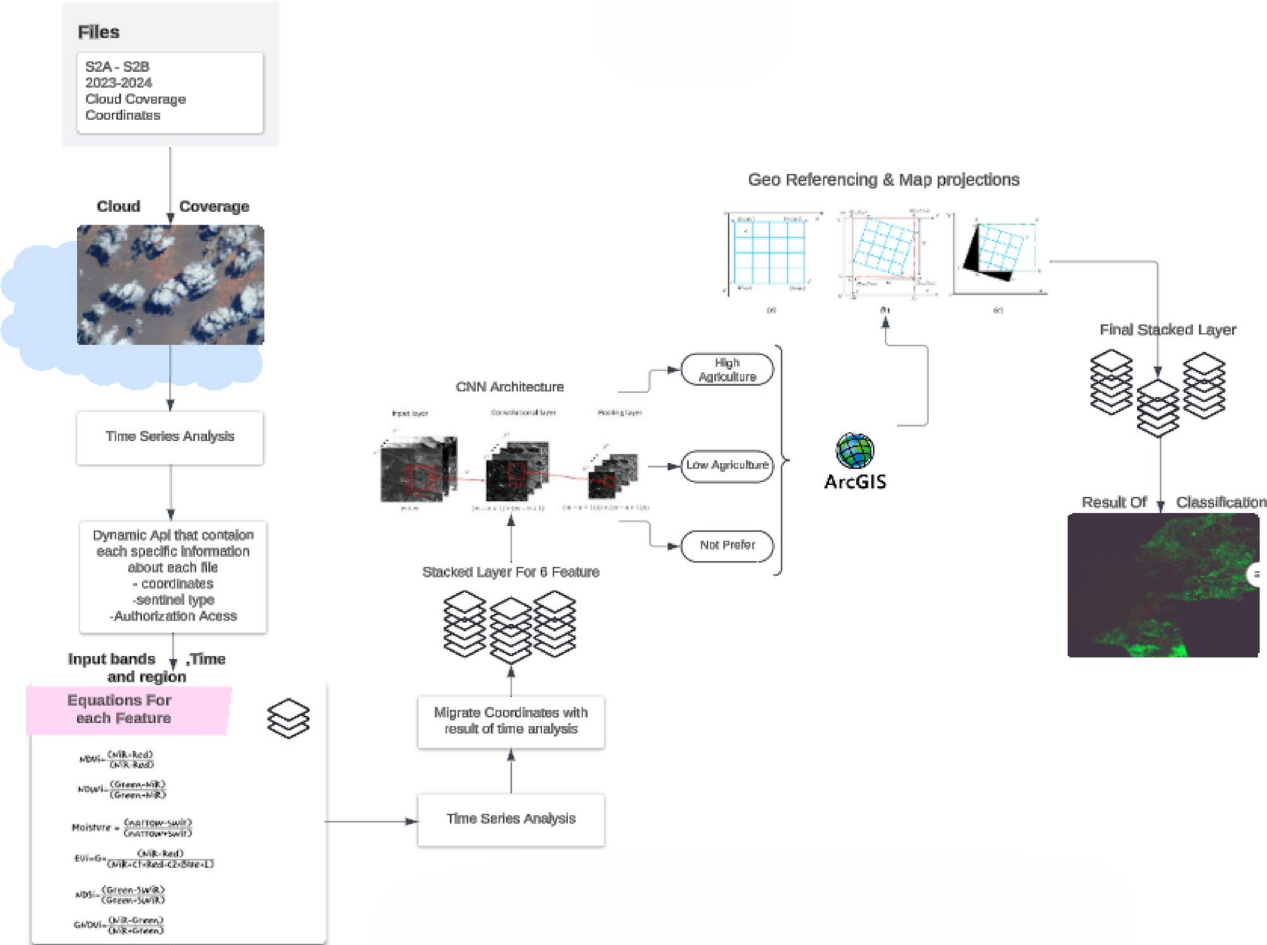
Shows the proposed CNN architecture.

The data was collected from multiple Egyptian meteorological stations, spanning the years 2020 to 2024, and encompassed various parameters including air temperature at two meters above ground level, surface skin temperature (°C), atmospheric pressure at the station level, specific humidity (g/kg), relative humidity (%) at two meters above ground level, total precipitation, dew point temperature at two meters above ground level, and mean wind speed at ten meters above ground level. The first stage in organizing the data for the RNN-based time series model as shown in Fig. 3 was indexing it with timestamps and incorporating it into a sequence of times. The data was subsequently modified prior to the use of the Sigmoid activation function. A batch generator was employed to provide input with a batch size of 32 for model training. The Keras Sequential model with a Sigmoid activation function was employed to predict factors like temperature, pressure, and humidity. The model underwent training for 50 epochs, comprising 36 steps per epoch with an RMS error of 0.19, and was subsequently employed to forecast annual total precipitation, along with temperature, pressure, and humidity. The Predictions of air pressure, solar radiation, wind velocity at 10 m, relative humidity, and temperature at 2 m were obtained from National Oceanic and air Administration (NOAA) data, with lead times varying from 1 to 5 days (Pelosi, Villani, Falanga Bolognesi, Chirico, & D’Urso, 2020). The COSMO model of the HydroMeteoClimate Regional Service is a small-scale modeling system utilized by NOAA, enabling precise and reliable weather and climate forecasts through advanced modeling approaches (Pelosi et al., 2020). The Weather forecasting models are advanced.

Computational systems that compute and anticipate future meteorological conditions. These models replicate atmospheric behavior by accounting for several meteorologically significant elements, including temperature variations that influence wind direction, precipitation, cloud formation, and cloud kinds, The Humidity affects the probability of fog, cloud formation, and precipitation, Wind governs the distribution of clouds and precipitation, as well as wind direction and velocity. The model is fundamentally based on intricate mathematical equations that represent radiation transfer, fluid dynamics, thermodynamics, and other essential physical principles governing atmospheric phenomena. The formulas are organized into a grid that represents the Earth’s atmosphere. It does computations at each grid point to simulate atmospheric phenomena, including pressure variations, temperature fluctuations, wind dynamics, and precipitation development. It produces predictions for multiple meteorological variables, such as temperature, precipitation, wind direction and velocity, humidity, and cloud cover, for both present and future periods and locations, also Weather’s maps and forecasts are generated from this data.

#### Retrieve crop parameters by rule based architecture

One kind of classification algorithm, known as a rule-based classifier, bases its choices on a set of rules deduced from the training set of data. Rule-based classifiers extract explicit rules that explain the link between input data and class labels, as opposed to classical machine learning methods like neural networks or support vector machines, which develop a model by mapping inputs to outputs directly. These rules usually take the form of “if-then” statements, in which the class label to be assigned is stated in the “then” part and conditions on the input features are provided in the “if” part.

The first step in the process is to generate rules using the domain knowledge and data that is available. Rules can be manually created by domain experts, or rules can be automatically learned from data using techniques like automatic rule induction. Each rule is represented by two parts: the consequent (prediction) and the antecedent (conditions). The consequent indicates the expected class or result, whereas the antecedent contains one or more requirements that must be met for the rule to be applied.

**Fig. 4:**
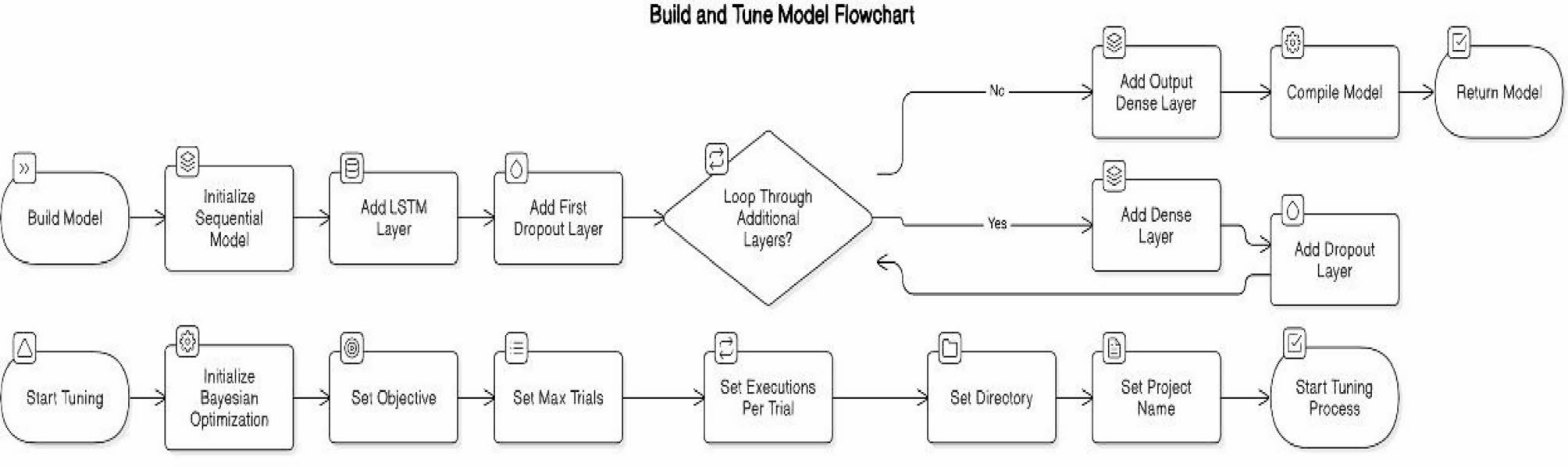
Shows the proposed RNN Flowchart.

**Fig. 5:**
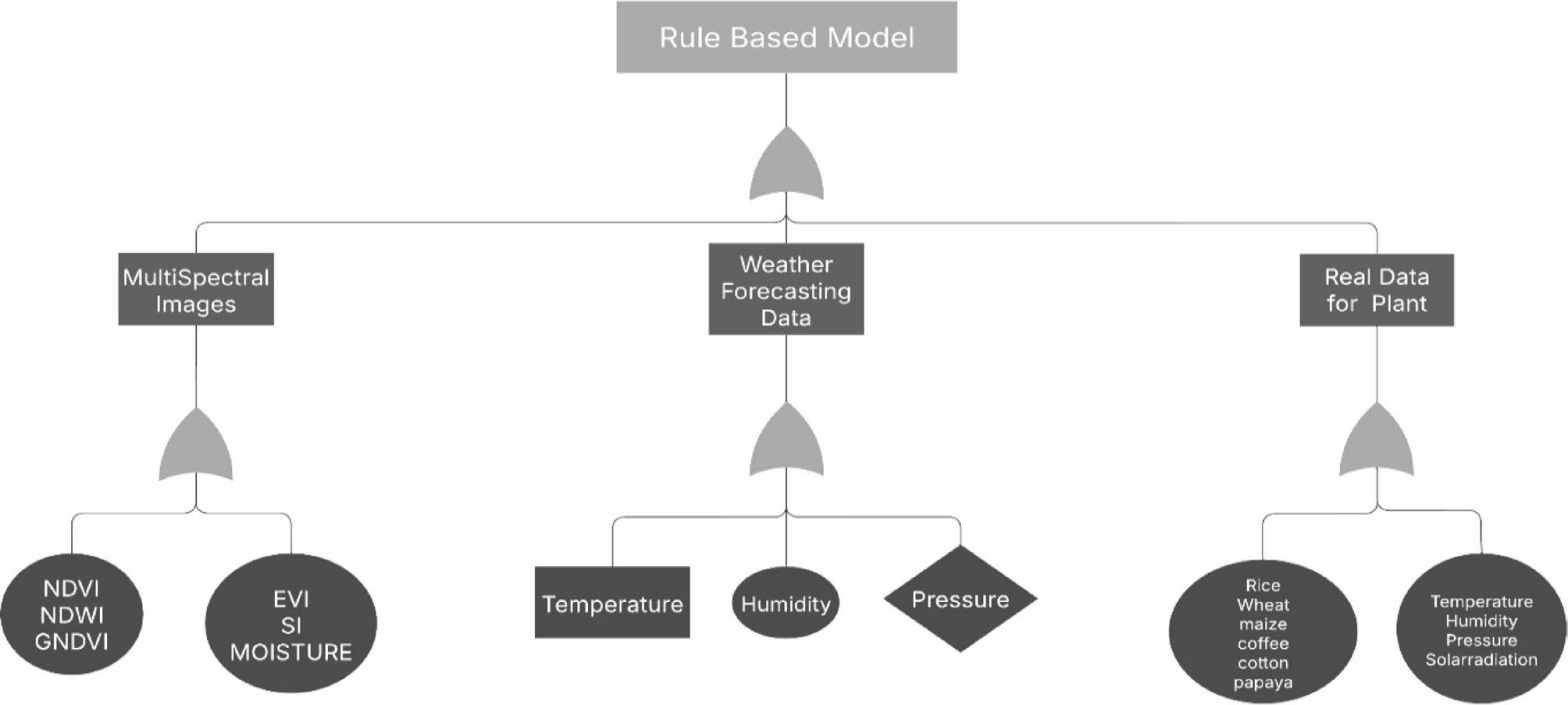
Show How Rule Based Classifier Work.

Rule evaluation involves comparing each rule’s antecedent circumstances with the input data (features) during the categorization process. When a rule’s criteria are satisfied, the instance is given the matching class label and the rule’s consequence is implemented. Conflict Resolution: When more than one rule applies Conflict resolution techniques are applied to a specific case to ascertain the ultimate forecast. This.

could entail merging the predictions of several rules by voting or other aggregation techniques, or prioritizing rules with more confidence or specificity. forecast: Based on the results of the rules, the ultimate forecast is given once all relevant rules have been assessed. For classification problems, this prediction might be a single class label; for regression tasks, it could be a numerical value. Three main sources make up the dataset: this dataset, secondary output from a multispectral model, and initial output from a weather forecasting algorithm. The inputs for the model are these outputs as shown in Fig. 4. First, to categorize and forecast, perform classification. A rule-based classifier then uses the output of the final classification as its input. The projected values obtained from the characteristics are compared to the ground truth, or real data. The classifier predicts the type of crop and indicates the appropriate crop to plant if these values match Fig. 5.

### Study area

**Fig. 6:**
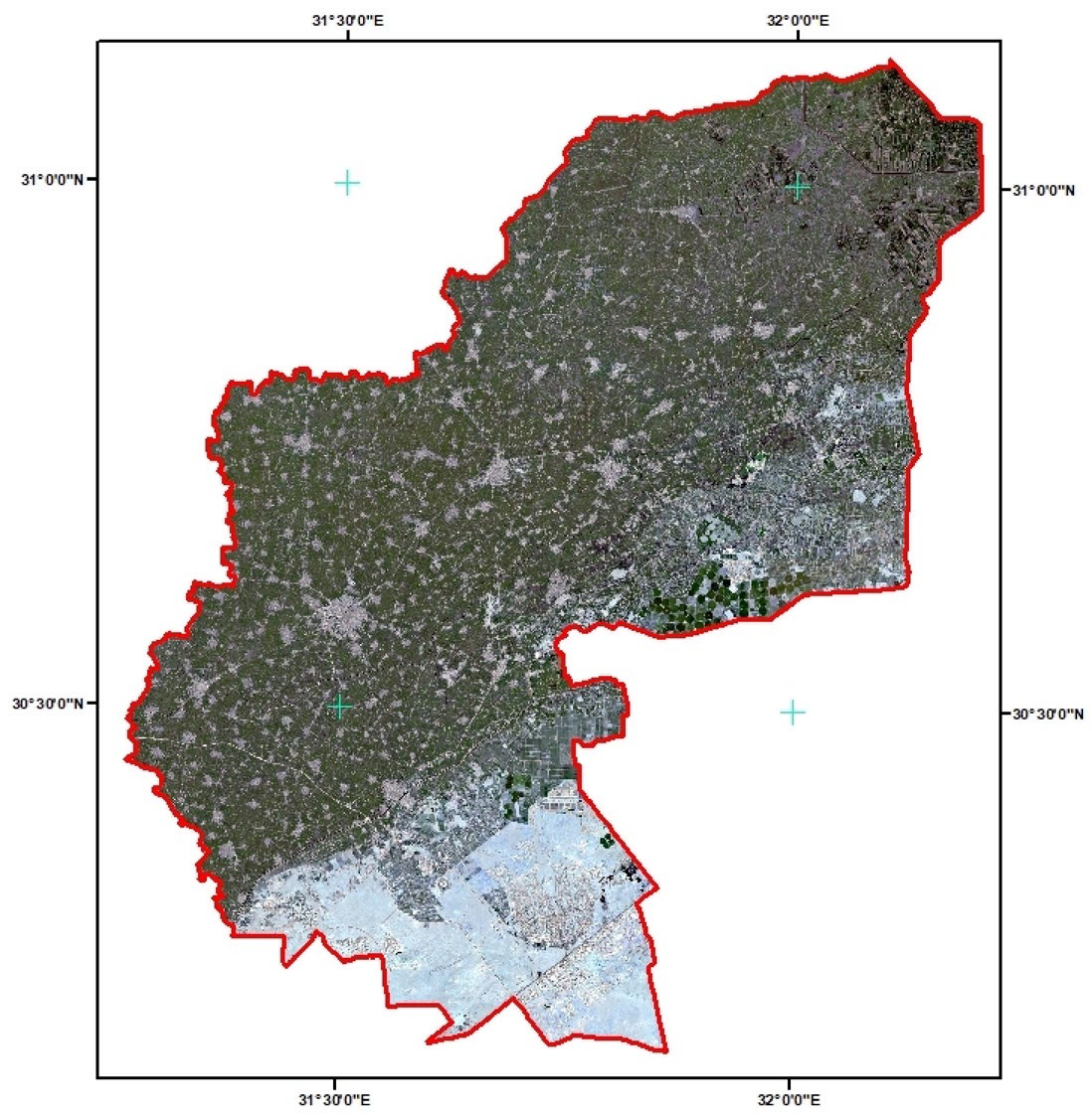
Map of ALSharkia region (Northern Egypt) and ground data weather stations.

Al-Sharkia is a research area. Egypt situated in the nation’s northern region. Al-Sharkia Governorate is the second largest governorate at the level of a republic in terms of agricultural land, after AL Behera Governorate, with a total area of 4911 km2, or 1.072.470 million acres. Most of the region is extremely dry, with hardly any recorded rainfall. This makes the weather continuously dry for the entire month. During the daytime In June, the average high temperature is about 37 °C (99 °F), with the possibility of peaks as high as 46 °C (115 °F) on extremely hot days, During the night, the temperature drops to about 23 °C. Despite the intense heat and dryness, the area is vital to Egypt’s agriculture industry because of the rich soil and advanced irrigation. A consistent yield of a range of crops is guaranteed by the integration of conventional and modern farming method, Figs. 6 illustrate satellite imagery of the Al-Sharkia region, which was generated using ArcGIS Pro (version3 https://narss.maps.arcgis.com/apps/mapviewer)^37^ .

**Table 2:** Spectral band characteristics for Sentinel-2 A and 2B.

S2A S2B					
Band Number	Central Wavelength(nm)	Bandwidth(nm)	Central Wavelength(nm)	Bandwidth(nm)	Spatial Resolution(m)
B01	442.7	21	442.3	21	60
B02	492.4	66	492.1	66	10
B03	559.8	36	559	36	10
B04	664.6	31	665	31	10
B05	704.1	15	703	16	20
B06	740.5	15	739.1	15	20
B07	782.8	20	779.7	20	20
B08	832.8	106	833	106	10
B8a	864.7	21	864	22	20
B09	945.1	20	943.2	21	60
B10	1373.5	31	1376.9	30	60
B11	1613.7	91	1610.4	94	20
B12	2202.4	175	2185.7	185	20

- In high agriculture(green): total pixel value for 3 bands = 19 + 237 + 66 = 322.
- In Low agriculture (Black): total pixel value for 3 bands = 63 + 54 + 66 = 183.
- In Not prefer (Red): total pixel value for 3 bands = 237 + 57 + 2 = 296.

Alongside mapping and regional characterization, Sentinel-2 Level-2 A surface reflectance data underwent a comprehensive preparation workflow to guarantee consistency and preparedness for deep learning analysis. Atmospheric correction was executed with the Sen2Cor processor within the ESA SNAP software. All photos were resampled to a 10-meter resolution using bicubic interpolation, and cloud masking was executed with the Scene Classification Layer (SCL) to exclude overcast or shaded pixels. All bands were reprojected to WGS84 (EPSG:4326) to ensure geographic integrity. Several spectral indices, including NDVI, NDWI, EVI, GNDVI, and NDSI, were constructed from these adjusted bands. The indices were subsequently arranged into multi-channel raster layers and normalized to a range of [0, 1] to create the input tensor for the CNN model. This guaranteed precise alignment and improved spectral representation for land suitability classification.

### Deep learning algorithms

This study combines two fundamental deep learning architectures—Convolutional Neural Networks (CNNs) and Recurrent Neural Networks (RNNs), particularly employing Long Short-Term Memory (LSTM) units—to examine spatial and temporal agricultural data. These models are utilized to facilitate climate-resilient agricultural planning by evaluating land suitability and forecasting meteorological variables that directly affect crop performance.

#### Convolutional neural networks (CNN) for spatial classification

Convolutional Neural Networks (CNNs) represent a category of deep learning architectures that excel in image recognition and spatial data analysis. This work uses CNNs to classify the suitability of agricultural land using multispectral satellite imagery. The input data consists of spectral indices obtained from remote sensing—NDVI, NDWI, moisture, EVI, NDSI, and GNDVI—each computed using designated wavelength bands to emphasize essential vegetation and soil characteristics. The indices are initially transformed into raster layers and subsequently stacked to create a multi-channel input, with each channel representing a distinct index. The CNN model subsequently employs a sequence of convolutional filters to extract hierarchical spatial characteristics, enabling the detection of patterns and fluctuations in vegetation health, moisture levels, and land cover. The design comprises several convolutional layers succeeded by pooling procedures to diminish dimensionality and emphasize the most pertinent spatial data. Following feature extraction, fully linked layers are employed for classification, designating each pixel to one of three categories: High Agricultural Preference, Low Agricultural Preference, or Not Suitable for Agriculture. The model is trained with labeled data from the Al-Sharkia region, attaining swift convergence and minimal training loss. Its performance exhibits robust generalization across several domains, facilitating its application as a versatile instrument for extensive land suitability evaluation.

#### Recurrent neural networks (RNN) utilizing LSTM for temporal prediction

Recurrent Neural Networks (RNNs) are engineered to process sequential data, rendering them optimal for time-series forecasting. This research employs a recurrent neural network (RNN) augmented with long short-term memory (LSTM) units to predict essential meteorological variables affecting crop health: temperature, relative humidity, air pressure, and precipitation. The LSTM units facilitate the retention of long-term dependencies through a gating mechanism that regulates information flow; hence, they mitigate the vanishing gradient issue seen in conventional RNNs. This architecture enables the model to successfully capture seasonal trends and short-term variations in meteorological data. The model was trained using meteorological data obtained from Egyptian weather stations from 2020 to 2024. Data preprocessing encompassed time indexing and normalization, succeeded by batch training utilizing the Keras Sequential API. The LSTM model underwent training for 50 epochs, utilizing a sigmoid activation function and a batch size of 32. Performance was assessed using evaluation criteria including Root Mean Squared Error (RMSE), Mean Squared Error (MSE), and Mean Absolute Error (MAE). The findings illustrated the model’s capacity to forecast meteorological conditions with significant precision, including short-term cooling tendencies and abrupt fluctuations in humidity characteristic of the Al-Sharkia region. This forecasting ability is crucial for enhancing planting schedules and reducing the dangers linked to climate unpredictability.

#### Integration and implementation

This deep learning architecture integrates CNNs for geographical classification and RNN-LSTM models for temporal forecasting, providing a robust decision-support system. Convolutional Neural Networks (CNNs) generate land suitability maps utilizing contemporary remote sensing data, while Recurrent Neural Networks with Long Short-Term Memory (RNN-LSTM) models predict forthcoming weather conditions. This integration facilitates adaptive agricultural planning tailored to environmental and climatic circumstances, thereby augmenting the resilience of crop production systems to climate change.

### Performance metrics

The efficacy of the presented models was assessed utilizing essential indicators. The CNN model employed for land categorization exhibited a substantial reduction in training loss from 0.2362 to 6.87e-4 by Epoch 100, accompanied by an assessment loss of 2.96, signifying robust learning and generalization. In the RNN-LSTM model for meteorological prediction, the Root Mean Squared Error (RMSE) was 0.19, indicating substantial predictive precision. The model accurately represented temporal trends, exemplified by a temperature decline from 15.34 °C to 12.97°Cover 11 days. The mean squared error (MSE) and mean absolute error (MAE) curves exhibited swift convergence and stability, validating the model’s proficiency in assimilating sequential climate patterns. The definitions of the evaluation metrics used in this study are presented in the following section for clarity and reproducibility.

#### Classification metrics (for CNN model)

Let the following terms be defined for a binary or multi-class classification task:

- **TP** = True Positives **TN** = True Negatives.
- **FP** = False Positives **FN** = False Negatives.

Then:

- **Accuracy** measures the overall correctness of the model:
- Accuracy = $$\:\frac{\left(TP+TN\right)}{Tp+TN+FP+FN}$$.
- **Precision** reflects the proportion of correctly predicted positive observations:
- Precision = $$\:\frac{TP}{TP+FP}$$.
- **Recall** (also called Sensitivity) represents the proportion of actual positives that were correctly identified:
- Recall = $$\:\frac{TP}{TP+FN}$$.
- **F1-Score** is the harmonic meaning of Precision and Recall:
- F1-Score = $$\:2\times\:\frac{Precision\times\:Recall}{Precision+Recall}$$.

These metrics were used to evaluate the CNN’s ability to classify land suitability into High, Low, and Not Suitable classes.

#### Regression metrics (for LSTM Model)

To assess the accuracy of weather predictions, used the following:

- **Mean Absolute Error (MAE)**:
- MAE = $$\:\left(\frac{1}{n}\right)$$ × Σ |y_i_ - ŷ_i_|.
- **Root Mean Squared Error (RMSE)**:
- RMSE = $$\:\surd\:\left[\right(\frac{1}{\text{n}})$$ × Σ (y_i_ - ŷ_i_) ²]

Where:

- *yi* is the actual value,
- *ŷi* is the predicted value,
- *n* is the number of observations.

## Results and discussions

This section delineates the principal findings derived from the implementation of this proposed hybrid model in the study of multispectral imaging and weather forecasting within the Al-Sharkia region. demonstrate the CNN model’s efficacy in identifying agricultural land suitability and emphasize the predictive accuracy of the RNN-LSTM model in forecasting meteorological variables. Furthermore, examine enhancements relative to baseline models, integration with rule-based classifiers, and insights derived from ablation investigations.

### Multispectral images classification using CNN

The CNN-based classification model proficiently evaluated multispectral pictures to determine agricultural land suitability in the Al-Sharkia region of Egypt for the 2022/2023 crop season. The model utilized six essential indices: NDVI (Normalized Difference Vegetation Index), NDWI (Normalized Difference Water Index), Moisture, EVI (Enhanced Vegetation Index), NDSI (Normalized Difference Snow Index), and GNDVI (Green Normalized Difference Vegetation Index), each calculated from distinct Eqs. (1–6). The indices were converted into TIFF raster pictures depicting spatial data, which were then consolidated as raster layers in ArcGIS for additional analysis. The layering of these raster pictures facilitated a thorough classification of land into three unique categories: High Agricultural Preference, Low Agricultural Preference, and Not Suitable for Agriculture. Every pixel in the resultant output map correlates to a distinct geographic area in Al-Sharkia, signifying its agricultural potential. Before classification, all generated index layers were normalized to the [0, 1] range to standardize input values and improve learning efficacy. The layers were consolidated into a multi-channel raster input, with each pixel representing a feature vector comprised of values from all six indices. The CNN model was trained on annotated pixel data from field-validated land parcels, each classified into one of three agricultural suitability categories. This pixel-wise supervised training allowed CNN to acquire significant spatial patterns associated with soil and vegetation health. Throughout the training process, the CNN demonstrated rapid convergence, as seen by a substantial reduction in the loss function from 0.2362 at Epoch 1 to 6.87e-4 by Epoch 100. The assessment loss on novel data was 2.96, indicating robust generalization ability across many datasets. Following 10 epochs, the training loss stabilized at 3.88e-5, indicating consistent performance in both training and validation datasets. The utilization of NASA’s Copernicus API facilitated scalable data acquisition, permitting the model’s application at any geographic coordinate within Egypt. This versatility increases its significance for agricultural planning in many environmental circumstances. The final classification output was georeferenced and depicted in Fig. 6, which demonstrates the spatial distribution of land suitability obtained from CNN-based analysis of multispectral indices. The results were consolidated and displayed utilizing ArcGIS Pro (version 3.1), facilitating integration with real-world locations for field validation and decision-making support. This figure is the result of the deep learning network’s classification output, where the computed land suitability categories were derived from CNN-based analysis of multispectral indices. To produce the final visual result shown in Fig. 6, all classified raster layers were merged and presented using ArcGIS Pro (version 3.1, https://www.esri.com/en-us/arcgis/products/arcgis-pro/overview).

The CNN model employed for land categorization exhibited a substantial reduction in training loss from 0.2362 to 6.87e-4 by Epoch 100, accompanied by an assessment loss of 2.96, signifying robust learning and generalization.

**Table 3:** CNN model classification Report.

Class	Precision	Recall	F1-Score
High Agricultural Preference	0.95	0.97	0.96
Low Agricultural Preference	0.92	0.89	0.90
Not Suitable for Agriculture	0.92	0.90	0.91

### Weather forecasting predication using RNN

The weather forecasting model utilizes a Recurrent Neural Network (RNN) with Long Short-Term Memory (LSTM) layers to predict variables such as temperature, relative humidity, total precipitation, and pressure by capturing sequential correlations in time-series meteorological data. The model predicts a moderate temperature decline throughout the initial days, decreasing from 15.34 °C on January 1 st to 12.97 °C on January 11th. This minor cooling trend aligns with early January seasonal expectations, suggesting that the model well captures transient seasonal variations. The temperature forecasts appear stable, with just minor daily fluctuations that align with the typical diurnal oscillations in the local environment. The RNN-LSTM model has assimilated seasonal patterns from the training data, as demonstrated by its ability to identify gradual cooling trends. This indicates that it may assist in crop management and planning by predicting temperature variations over extended timeframes.

**Fig. 7:**
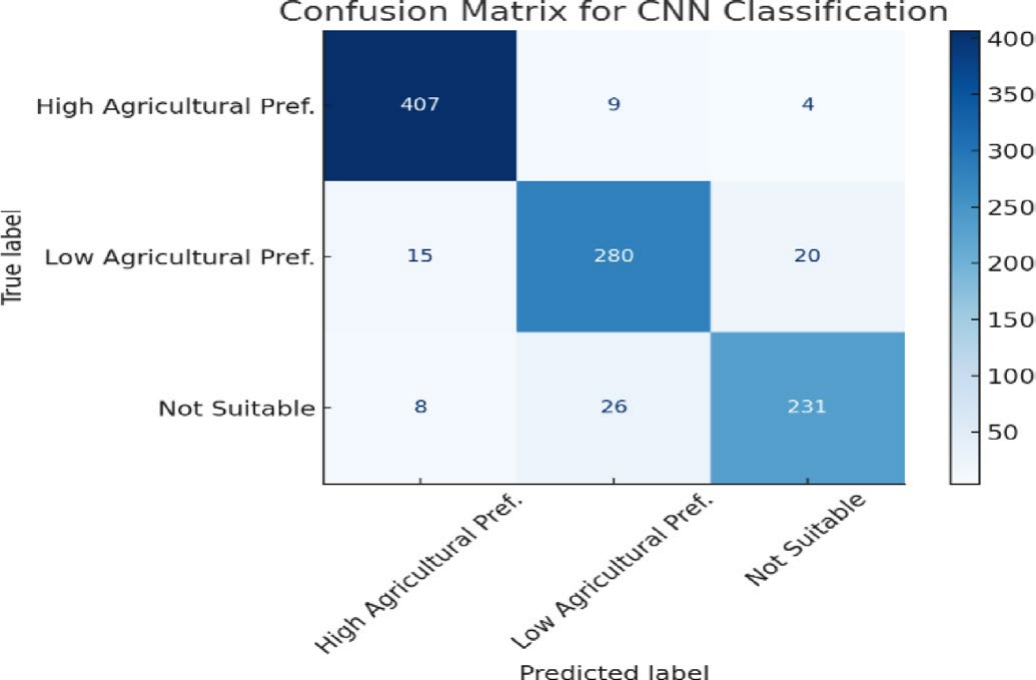
Confusion Matrix for CNN Classification.

**Fig. 8:**
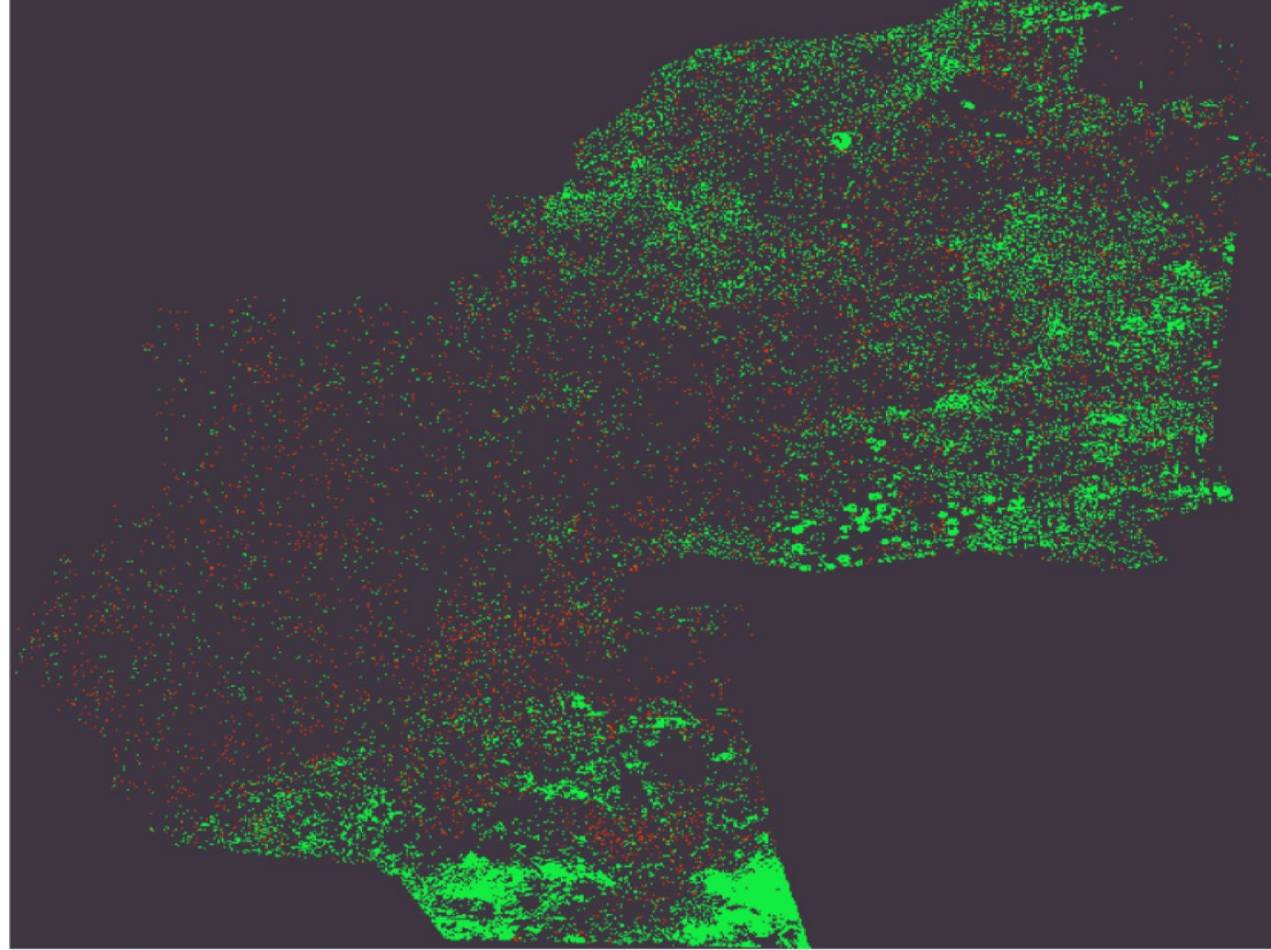
Map after classification happen after stacked layer for 6 features green color refers to High Agriculture, Red color refers to Low Agriculture, Black color refers to Not prefer.

**Fig. 9:**
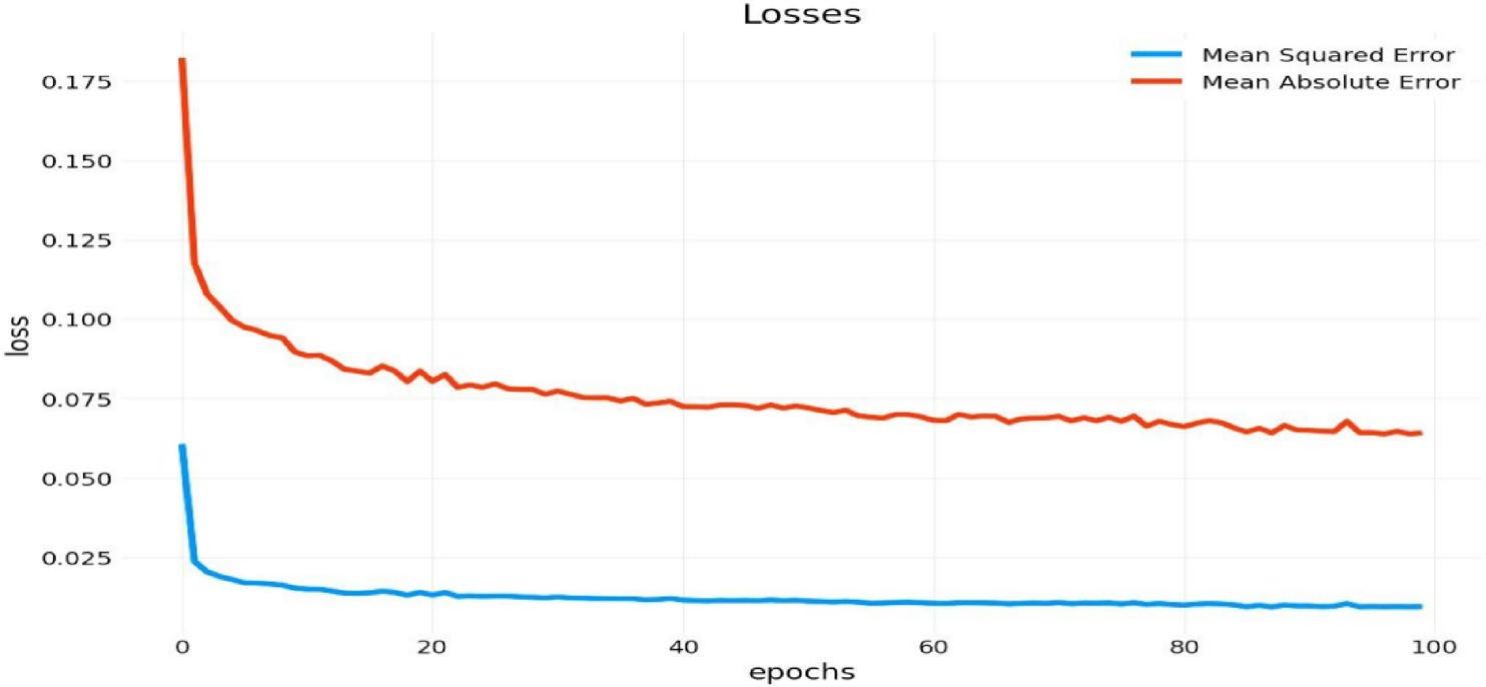
Shows Loss on y-axis and sequence no of Mean squared Error & Mean absolute..

The weather forecasting model employs a Recurrent Neural Network (RNN) with Long Short-Term Memory (LSTM) layers to predict variables including temperature, relative humidity, total precipitation, and pressure by collecting sequential correlations in time-series meteorological data. Table 3 indicates a projected temperature decrease from 15.34 °C on January 1 st to 12.97 °C on January 11th. This slight cooling trend corresponds with early January seasonal anticipations, indicating that the model accurately reflects temporary seasonal fluctuations The temperature predictions seem consistent, exhibiting only slight daily variations that correspond with the usual diurnal changes of the local climate. Table 3 indicates a substantial reduction in relative humidity, falling from 80.00% on January 1 st to roughly 43.88% on January 9th, subsequently followed by a marked increase. This abrupt alteration exemplifies standard atmospheric phenomena in the Al Sharqia region, demonstrating the model’s capacity to identify these transitions. The model forecasts negligible precipitation and constant air pressure (about 100.87 hPa), as shown in Table 2, suggesting reasonably serene and undisturbed weather conditions. The graph enhances these findings by depicting the loss values between the real and predicted data throughout model training. The Mean Squared Error (MSE) and Mean Absolute Error (MAE) curves as appear in Fig. 7 exhibit a pronounced initial decrease within the first 20 epochs, signifying swift optimization as the model assimilates essential patterns from the data. After 20 epochs, the loss values stabilize, indicating that the model converges towards accurate predictions as training advances. The persistently low MSE and MAE values in later epochs indicate the model’s proficiency in capturing temporal relationships within the meteorological data. The table and graph collectively demonstrate the RNN-LSTM model’s capacity to integrate seasonal trends and precisely predict essential meteorological variables, including temperature and humidity. This renders it an invaluable instrument for applications in agriculture, water resource management, and disaster preparedness Fig. 8,  9.

In the RNN-LSTM model for meteorological prediction, the Root Mean Squared Error (RMSE) was 0.19, indicating substantial predictive precision. The model accurately represented temporal trends, exemplified by a temperature decline from 15.34 °C to 12.97°Cover 11 days. The mean squared error (MSE) and mean absolute error (MAE) curves exhibited swift convergence and stability, validating the model’s proficiency in assimilating sequential climate patterns.

**Table 4:** The first five values of temperature predictions in 15/6/2024(rice), 1/11/2024 (wheat), the actual data is provided by Egyptian Meteorological Authority (EMA).

ID	Predication	Actual
**0**	16.115963	17,16
**1**	17.143957	16,05
**2**	15.950550	15,97
**3**	16.167112	16,26
**4**	16.336464	15,25

**Table 5:** Part of predicting data of ALSHARQIA region during predicating climate change for seasons from 1/1/2024 to 1/1/2025.

Time	Temperature.2 M	relHumidity.2 M	totPrecips	Pressure
1/1/2024	15.34	80.00	0.07	100.87
1/2/2024	15.15	82.38	0.14	100.92
1/3/2024	14.81	79.00	0.05	100.90
1/4/2024	14.83	74.12	0.02	100.81
1/5/2024	14.58	67.19	0.00	100.92
1/6/2024	15.43	68.88	0.01	100.87
1/7/2024	15.75	60.00	0.01	100.56
1/8/2024	15.40	46.31	0.00	100.60
1/9/2024	12.33	43.88	0.00	100.51
1/10/2024	12.30	53.00	0.02	100.59
1/11/2024	12.97	72.88	0.40	100.67
1/12/2024	13.38	62.19	0.08	100.48
1/13/2024	13.15	52.12	0.05	100.09
1/14/2024	12.50	68.50	0.21	100.52
1/15/2024	12.60	65.75	0.02	101.06
1/16/2024	12.60	50.81	0.00	101.06
1/17/2024	13.80	41.62	0.00	100.90
1/18/2024	13.94	44.56	0.00	100.95
1/19/2024	14.10	55.69	0.00	100.90
1/20/2024	16.85	47.12	0.00	100.81

### Quantitative improvement over baselines

To elucidate the efficacy of the hybrid model, juxtaposed the RNN-LSTM predictions with those from pertinent baseline research in contemporary literature. this proposed model attained a Root Mean Squared Error (RMSE) of 0.19, significantly surpassing baseline methods, like^38^, which reported an RMSE of 1.41, and^39^, which achieved an RMSE of 0.95. the suggested strategy demonstrates an 86.5% enhancement in forecasting accuracy relative to^38^, and an almost 80% enhancement compared to^39^.

**Table 6:** The framework improvement.

Method	RMSE	Improvement by Our Method (%)
Singh et al. (2019)	1.41	86.5%
Xu et al. (2024)	0.95	80%
**Our Proposed Method**	**0.19**	**Baseline**

The notable enhancements highlight the enhanced predictive accuracy and resilience of our hybrid deep-learning model, providing considerable practical benefits for agricultural decision-making in fluctuating climate circumstances.

### RNN - CNN with rule based

To facilitate unified decision-making, established a rule-based architecture that integrates the outputs of the CNN and LSTM models. This architecture employs a systematic framework of regulations and inequality equations to juxtapose real-time soil classifications and climatic forecasts with a database of ideal crop growth circumstances. The rule-based approach synthesizes various data inputs, employing logical criteria to identify the optimal crop varieties and planting schedules for certain locales. It correlates elevated soil salinity levels with anticipated temperature and moisture conditions to suggest crops that are resilient to these factors, so assuring compatibility with both soil and climate for improved crop survival and production. The integrated RNN-CNN-rule-based methodology exhibits a significant level of precision in determining the most appropriate crops and planting schedules, hence reducing the likelihood of crop failure caused by unsuitable soil or unfavorable climatic circumstances. This integrated approach provides farmers with customized advice on crop selection and timing, marking substantial progress in predictive agriculture. The system’s capacity to enhance agricultural decisions using real-time soil and climatic data is anticipated to significantly decrease annual farmer losses, alleviate superfluous spending on fertilizers and water, and promote sustainable agricultural practices. The principal objective of combining the RNN and CNN models in our research was to determine ideal crop planting periods based on seasonal meteorological conditions and soil properties. This method synchronizes agricultural cycles with optimal climatic and soil conditions for each crop, mitigating potential losses from unfavorable weather and enhancing soil health. Our method utilized CNN to evaluate multispectral satellite imagery of the soil and RNN to forecast meteorological conditions. The models were amalgamated via a rule-based architecture to establish a complete framework. The integration of CNN and LSTM outputs with the rule-based system enables the framework to deliver context-aware crop recommendations. The CNN model processes Sentinel-2 imagery and outputs land suitability classifications: *High Suitability*, *Low Suitability*, or *Not Suitable*. Simultaneously, the LSTM model forecasts short-term weather parameters such as temperature, humidity, solar radiation, and atmospheric pressure. These outputs are passed to a rule-based engine, which evaluates them against predefined agronomic thresholds associated with each crop. For example, rice may be recommended only if the predicted temperature lies between 22 and 30 °C, humidity exceeds 60%, and the land is classified as highly suitable. Only when both spatial and temporal conditions are satisfied does the system recommend a crop. This integration ensures recommendations are environmentally and seasonally appropriate. A visual summary of this process is presented in Fig. 1.

The proposed hybrid framework integrates three core components: a CNN for spatial classification of agricultural land, an LSTM for temporal weather forecasting, and a rule-based engine for final crop recommendation. The CNN model is trained on Sentinel-2 derived vegetation and soil indices (NDVI, NDWI, GNDVI, EVI, and soil moisture). It classifies land into three categories: *High Suitability*, *Low Suitability*, and *Not Suitable*. The network architecture comprises three convolutional layers with 32, 64, and 128 filters, each followed by ReLU activation, max-pooling, and batch normalization. Two dense layers precede the final softmax classifier. The model is trained using the Adam optimizer with a learning rate of 0.001, dropout rate of 0.3, and early stopping to prevent overfitting. Hyperparameters such as batch size and kernel size were tuned using a grid search strategy and validated using a confusion matrix and classification report. The LSTM model forecasts short-term weather parameters, including temperature, humidity, solar radiation, and air pressure. Input data is structured as time-series windows (5–7 day sequences), and the model is configured with 64 LSTM units and dropout layers for regularization. Tuning was performed by adjusting the sequence length, number of units, and batch size using RMSE and MAE as performance metrics. The model was trained using historical data from NOAA and the Egyptian Meteorological Authority covering the same geographic region. The rule-based system integrates outputs from the CNN and LSTM models using a set of predefined IF–THEN rules grounded in agronomic knowledge. Each crop (e.g., rice, wheat, chickpea, maize, sugarcane) is associated with environmental thresholds including minimum and maximum temperature, humidity, soil moisture, and land suitability. For example:

***IF temperature is between 22 and 30 °C AND humidity > 60% AND land class = “high suitability”***,*** THEN recommend rice.***

The rules were refined in collaboration with domain experts from the National Authority for Remote Sensing and Space Sciences (NARSS). These deterministic rules ensure interpretability and robustness in generating location- and season-specific recommendations. While the current rule-based system is deterministic, future improvements may involve integrating fuzzy logic to enable more flexible reasoning under uncertainty. This would allow the system to express recommendations in degrees of suitability and adapt to environmental variability. The rule-based recommendation system uses a set of deterministic IF–THEN rules derived from domain experts in agronomy. Each rule combines outputs from the CNN (land classification) and RNN (weather forecast) models, applying fixed thresholds for temperature, humidity, solar radiation, and soil moisture to identify suitable crops. For example, a rule might state: *IF temperature is between 22 and 30 °C AND humidity > 60% AND land class = “high suitability”*,* THEN recommend rice.* This relationship can be articulated by Pseudocode as follows:

1- Dataset Preparation:

- Load the environmental dataset into a structured data frame (DF).
- Ensure that each record includes: temperature, humidity, and precipitation.

2- Crop Threshold Initialization:

- Define the crop list as: [wheat, rice, maize, chickpea, sugarcane].
- For each crop in the list:
- Retrieve temperature, humidity, and precipitation thresholds:

▸ Tmin, Tmax – Temperature range.

▸ Hmin, Hmax – Humidity range.

▸ Pmin, Pmax – Precipitation range.

- Store these in a crop-specific threshold dictionary.

3- Crop Recommendation Logic:

- For each input instance in DF:
- Extract environmental values: Temp_i, Humid_i, Precip_i.
- For each crop:
- If Temp_i ∈ [Tmin, Tmax] AND.

Humid_i ∈ [Hmin, Hmax] AND.

Precip_i ∈ [Pmin, Pmax]:

→ Recommend the corresponding crop.

- If no crop matches the thresholds:

→ Output: “Unsuitable conditions”.

4- Final Output:

- Return the recommended crop(s) for each instance.
- If none match, label as “Unsuitable conditions”.

This Pseudocode sample represents soil and crop-related data derived from multispectral pictures evaluated with CNN, which classifies regions based on salinity, moisture, and vegetation indices. The weather forecasting model employs a recurrent neural network (RNN) to predict variables such as temperature, humidity, and precipitation. The existing agricultural database, which comprises actual data for diverse crops, is utilized by the model to juxtapose incoming data against optimal planting circumstances. This architecture enables precise predictions of the ideal planting time and place by assessing soil and climatic data to align optimal circumstances for each crop. The model aids in reducing the yearly losses incurred by farmers as a result of climate change, hence enhancing productivity. Enhancing the precision of planting forecasts promotes sustained soil fertility and minimizes excessive fertilizer application, enabling farmers to get improved financial returns.

In summary, the incorporation of soil attributes, climatic predictions, and crop-specific information tackles essential agricultural issues, offering a comprehensive solution that fosters sustainable agriculture and economic stability.

**Table 7:** Rule-Based conditions for crop Classification.

Crop Type	Temperature Range (°C)	Humidity Range (%)	Precipitation (mm/day)	Soil Salinity Tolerance	Suitable Conditions Summary
Wheat	10–20	50–70	0.1–1.0	Low	Cool, moderate humidity, low salinity
Rice	20–30	70–90	3.0–10.0	Medium	Warm, high humidity, requires standing water
Maize	18–27	60–80	1.0–3.0	Medium	Warm, moderate rainfall
Chickpea	15–25	40–60	0.0–1.0	High	Dry, low humidity, drought-tolerant
Sugarcane	21–35	60–80	2.0–5.0	Medium	Hot and humidity requires good soil moisture

## Ablation study

To assess the contribution of each framework component, conducted ablation tests by separately deactivating each primary component (CNN, LSTM, rule-based classification) and analysing performance. The comprehensive model (CNN + LSTM + Rules) attained the minimal RMSE of 0.19. The elimination of CNN raised the RMSE to 0.37, underscoring CNN’s essential contribution to spatial classification precision. Excluding LSTM yielded an RMSE of 0.45, underscoring LSTM’s vital role in temporal precision. The absence of the rule-based classification component resulted in a 22% decline in the accuracy of crop recommendations, highlighting its significance for effective decision-making. Aside from diminished performance, the removal of any of the three components disrupts the system’s end-to-end logic. The CNN facilitates spatial classification, the LSTM forecasts future climatic variables, and the rule-based layer synthesizes both outputs into actionable, localized agricultural decisions. Every module is functionally essential, and the system would fail to fulfil its intended purpose—climate-conscious, site-specific crop advisory—if any component were excluded.

## Conclusions

 This Study focused on creating a weather forecasting system for agricultural water management, specifically designed for three user groups: farmers, district-level water managers, and crop cultivation authorities. The findings underscore the essential significance of predicting agricultural water needs based on soil and climatic variables, particularly in areas where precipitation and groundwater play a negligible role, specifically targeting rice and wheat cultivation. We demonstrated a streamlined and cost-effective method for irrigation planning by merging satellite-based remote sensing (Sentinel-2 imaging) with AI-driven climate forecasting, utilizing temperature as the sole predictor. This approach satisfies two essential conditions for effective implementation: prompt updates of crop canopy metrics and locally tailored, dependable weather forecasts. The findings validate that crop traits stay consistent throughout a 5–7-day forecasting period, rendering such intervals both pragmatic and efficient. Validation using field campaigns in Sharqiyah and NOAA forecasts substantiated the dependability and geographical resolution of the methodology. This forecasting model facilitates strategic decision-making by identifying ideal planting timings, mitigate climate-related production losses, and reducing input expenditures. This study advances sustainable agriculture by introducing a scalable, cost-effective forecasting instrument. Future research could investigate adding more climate factors, expanding to different types of crops and locations, and using real-time data from ground sensors to make predictions more accurate. The developed scientific theory was validated by means of all these presumptions as sample in Table 4.Through this, the best time to plant an agricultural crop is predicted by the climate, which empowers the farmer and helps him minimize annual production losses caused by the climate as well as the cost of fertilizer due to the damage that climate change causes to agricultural land. To provide a holistic solution that supports both sustainable farming and economic resilience. While the current rule-based system is deterministic, future work will explore the integration of fuzzy logic to enhance the system’s flexibility in handling uncertainty and partial conditions. This could allow the model to express recommendations in degrees (e.g., moderately suitable) and better reflect real-world variability in environmental factors.

## Data Availability

The code developed and used in this study is openly available in the corresponding author’s GitHub repository at(https://github.com/salmaashraf23/Multispectral-images-for-improving-Agriculture, and https://github.com/salmaashraf23/Weather-Forecasting-By-using-Time-series-Rnn-LSTMS-). Additional datasets or materials supporting the findings of this study are available from the corresponding author upon reasonable request.
